# SiC Foams for the Photocatalytic Degradation of Methylene Blue under Visible Light Irradiation

**DOI:** 10.3390/ma16041328

**Published:** 2023-02-04

**Authors:** Karla Begonia Cervantes-Diaz, Martin Drobek, Anne Julbe, Julien Cambedouzou

**Affiliations:** Institut Européen des Membranes (IEM), Univ Montpellier, CNRS, ENSCM, Place Eugene Bataillon, 34095 Montpellier, France

**Keywords:** silicon carbide, preceramic polymers, SiC ceramic foam, photocatalysis

## Abstract

SiC foams were synthesized by impregnating preceramic polymer into polyurethane foam templates, resulting in a photo-catalytically active material for the degradation of methylene blue. The crystalline structure, electronic properties, and photocatalytic performance of the SiC foams were characterized using a series of experimental techniques, including X-ray diffraction, electron microscopy, energy dispersive X-ray spectroscopy, N_2_ physisorption measurements, UV-visible spectroscopy, and methylene blue photodegradation tests. The original polyurethane template’s microporous structure was maintained during the formation of the SiC foam, while additional mesopores were introduced by the porogen moieties added to the preceramic polymers. The prepared SiC-based photocatalyst showed attractive photocatalytic activity under visible light irradiation. This structured and reactive material offers good potential for application as a catalytic contactor or membrane reactor for the semi-continuous treatment of contaminated waste waters in ambient conditions.

## 1. Introduction

There is currently a major problem of water pollution caused by the uncontrolled release of dyes in rivers across the world. These organic pollutants not only cause serious environmental problems due to their toxicity but also harms humans by inducing carcinogenic, teratogenic, and mutagenic effects [1]. To minimize the risk of contamination and possible health troubles, these effluents should be treated before being released into the environment. In fact, once rejected, the effective remediation of these molecules becomes more complicated due to the huge volume of water to be treated, with low pollutant concentration.

Among different methods for pollutant abatement, photocatalytic degradation is an attractive and largely investigated approach [2,3,4,5]. It allows a complete degradation of organic pollutants at ambient temperature, with the advantage of forming non-toxic byproducts in the case of their total mineralization to water and CO_2_ [1]. The photocatalytic process is typically induced by the irradiation of a semiconductor, which conducts the excitation of free electrons (e^−^) in the valence band (VB). These electrons then migrate to the conduction band (CB), leaving holes (h^+^) in the VB [6], resulting in the production of electron-hole pairs that move to the surface of the semiconductor. The h^+^ and e^−^ then induce the production of hydroxide radicals (∙OH) and superoxide radicals (∙O_2_^−^), which are very reactive and promote the oxidation of organic pollutants into CO_2_ and H_2_O [7,8,9].

Several metal oxide materials with suitable properties such as TiO_2_ [10], ZnO [11], SnO_2_ [12], Fe_2_O_3_ [13], and V_2_O_5_ [14] can be used as photocatalysts, but most of them are only photoactive in the ultra-violet (UV) range, as their bandgap is larger than 3 eV [15]. Hence, they reach their maximum efficiency only when irradiated by UV lamps, which complicates the process technology and generates additional costs. The wavelength range can be eventually extended to the visible light region, e.g., for TiO_2_/CoFe_2_O_4_ [16] and TiO_2_/CoFe_2_O_4_/Ag nanocomposites [17], which in addition possess attractive magnetic properties for further processing.

Besides metal oxides, non-oxide materials or compounds associating oxides and carbon nitrides have shown attractive photocatalytic activity in the visible light region. For example, g-C_3_N_4_/SiO_2_ has demonstrated high photocatalytic activity for the degradation of 2,4-dichlorophenol and rhodamine B in the visible range [18]. A SiO_2_-TiO_2_/g-C_3_N_4_ composite was also studied for its ability to degrade rhodamine B, producing hydrogen under solar light conditions [19]. In another recent study, g-C_3_N_4_ quantum-dot-modified TiO_2_ nanofibers were found to degrade tetracycline in the presence of copper [20]. Several water pollutants have also been eliminated by using Ag-decorated TiO_2_/g-C_3_N_4_ nanocomposites [21]. Other materials such as SiC, featuring attractive physico-chemical properties [22,23], have also been investigated for the degradation of organic compounds in a water environment under UV light irradiation. For example, TiO_2_/β-SiC foams were used for the photocatalytic degradation of Diuron [6], while organic dyes (orange G and amido black 10B) have been successfully eliminated with the help of Ag-decorated β-SiC [24].

Moreover, SiC has also been investigated in photocatalytic reactions at higher wavelengths, taking advantage of its bandgap capability to absorb irradiation in the visible light range [15,25,26]. In fact, depending on its crystalline structure, carbon content, and presence of doping atoms, the SiC bandgap can be slightly shifted to lower values (typically 2.4 and 3.0 eV for a β-SiC and α-SiC, respectively [15]), thus reaching the domain of visible light. In this context, both SiC nanowires (synthesized by sol-gel carbothermal reduction) [27] and SiC hollow spheres with a bandgap of 2.15 eV (obtained by a vapor–solid reaction using carbon spheres as templates) [28] have been successfully tested for the degradation of methylene blue (MB).

In this work, we present the synthesis and photocatalytic performance of a SiC foam material prepared using recycled polyurethane foam (PU) as a solid template and a polycarbosilane (PCS) preceramic polymer as a SiC precursor. The PU template was impregnated with a PCS solution and mixed with a triblock copolymer serving as a porogen to generate mesopores in the final SiC material, according to the procedure already described in our previous work [29]. The microstructure and crystalline structure of the resulting material were determined by scanning electron microscopy (SEM) coupled with energy dispersive X-ray spectroscopy (EDX), X-ray diffraction, infrared spectroscopy, and nitrogen sorption measurements. The functional properties of the material were evaluated in terms of its photocatalytic efficiency for the removal of MB under visible light irradiation.

## 2. Materials and Methods

### 2.1. Materials

The polyurethane (PU) foam template was obtained from electronic device packaging wastes. The SiC precursor was a commercial allylhydridopolycarbosilane (AHPCS, SMP-10 Starfire Systems Inc., East Glenville, NY, USA) with structural formula (SiH_2_CH_2_)_0.9_(Si(allyl)HCH_2_)_0.1_. A commercial SBS block copolymer (polystyrene-block-polybutadiene-block-polystyrene, 30 wt%, MW = 140, Sigma-Aldrich, St. Louis, MO, USA) was used as a porogen. Cyclohexane C_6_H_12_ (ACS 99+ %, Alfa Aesar Haverhill, MA, USA) was used as a solvent.

For the photodegradation experiments, methylene blue (C_16_H_18_ClN_3_S · xH_2_O, 97+ %, Sigma-Aldrich) was used as a model organic dye.

### 2.2. SiC Foam Fabrication

SiC foam was synthesized by the so-called replica method, in which a polyurethane (PU) foam was used as a template. The template was then eliminated at high temperatures during the pyrolysis step. Prior to this, the PU foam was cleaned to remove impurities by being soaked in ethanol and rinsed with deionized water (repeated three times). The PU foam was then dried in an oven at 70 °C for 12 h under an air atmosphere.

The as-purified PU foam was then impregnated (for 30 min) with a SiC precursor solution prepared by dissolving the SBS triblock copolymer in cyclohexane, followed by the progressive addition of AHPCS under continuous stirring until the mixture was fully homogenized [29]. The mass ratio between AHPCS and copolymer was set at 1:1. The impregnated foam was dried for 30 min at room temperature before being placed into an alumina crucible and pyrolyzed in a tubular furnace under an argon atmosphere at 1200 °C for 2 h with a heating rate of 120 °C/h.

### 2.3. SiC Foam Characterization

The surface morphology of the foams was analyzed using a Hitachi S4800 scanning electron microscope (SEM) and energy-dispersive X-ray spectroscopy (EDX) was conducted on Zeiss EVO HD15 SEM. The crystalline structure of foams was characterized by X-ray diffraction on a Panalytical Xpert diffractometer using CuKα radiation as the X-ray monochromatic source. Absorbance spectra were obtained with a Jasco V-570 UV-Vis spectrophotometer in the wavelength range of 200–800 nm. FTIR spectra were obtained with a Nexus spectrometer in the range of 500–4000 cm^−1^. The optical bandgap was determined from absorbance data and using the absorption spectrum fitting method (ASF) [30]. Gas (nitrogen) sorption isotherms were recorded using a Micromeritics ASAP2020 apparatus. Specific surface area (SSA) was obtained by using the Brunauer–Emmett–Teller (BET) model and the pore size distribution was calculated by applying the Barret–Joyner–Halenda (BJH) method on the desorption branch.

### 2.4. Black Experiments and Photocatalytic Degradation under Visible Light Irradiation

**Figure 1:**
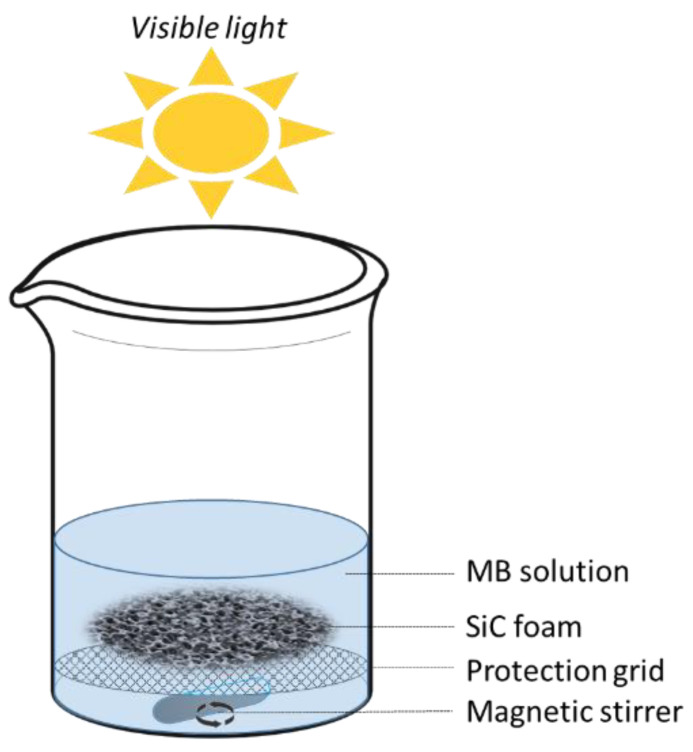
Setup used for the photocatalytic degradation of MB under visible light with SiC foam.

The photocatalytic degradation was carried out under visible light using an Osram HQI-TS 150 W lamp as an irradiation source. The concentration of methylene blue (MB) in the aqueous solution was set to 1.5 × 10^−5^ M, a value that is commonly used in relevant literature [28]. For each photocatalytic experiment, 80 mL of this solution was used and the SiC foam (typical size of 2 cm^3^) was added as a photocatalyst at a concentration of 20 g/L. The irradiation lamp was positioned directly above the solution and the walls of the beaker, which were obscured to prevent any potential contribution from natural light. The experimental setup is depicted in Figure 1.

The MB solution containing the SiC foam was first left under constant stirring in the absence of any light irradiation for 24 h to reach the adsorption equilibrium in order to assess the intrinsic adsorption capacity of the catalyst. After this period, the light was turned on and the solution was continuously irradiated for 8 h. An aliquot was taken every hour as a sample for quantifying the degradation of the dye. Each sample was filtered through a 0.2 μm filter and, subsequently, its absorbance (at λ = 664 nm) was measured using a Shimadzu UV-240IPC spectrophotometer. The degradation efficiency η of the SiC foam was calculated by applying the Beer–Lambert law, which states that the absorbance of MB is proportional to the concentration, as described in Equation (1) [31,32]:

(1) $$\eta \%=\frac{C_{0}-C}{C_{0}}\times 100\%=\frac{A_{0}-A}{A_{0}}\times 100\%$$

where $C_{0}$ and $A_{0}$ are the MB initial concentration (mg/L) and absorbance, and C and A are the concentration and absorbance at time *t*, respectively.

Then, the SiC foam was repeatedly rinsed with ethanol and water until the water was clear. The foam was then allowed to dry overnight at room temperature.

The adsorption capacity of SiC foam for dye removal was evaluated using experimental results obtained in the absence of light irradiation (blank experiments), applying the following Equation (2) [33]:

(2) $$q_{t}=\frac{(C_{0}-C)\times V}{m}$$

where $q_{t}$(mg/g) is the amount of MB adsorbed at time *t*, *V* is the volume of the solution, *m* is the mass of the used SiC foam, $C_{0}$ is the MB initial concentration, and *C* is the MB concentration at time *t*.

The percentage of MB dye retained on the SiC foam was calculated according to Equation (3) [34]:

(3) $$R\left(\%\right)=100-\left(\frac{C}{C_{0}}\times 100\right)$$

where *R* is the percentage of dye removal, *C* is the MB concentration at time *t*, and $C_{0}$ is the initial concentration at the beginning of the experiment.

## 3. Results

### 3.1. SiC Foam Characterization

The material resulting from the synthesis protocol described in the experimental section presents an open cell structure, similar in appearance to the PU foam used as a template.

**Figure 2:**
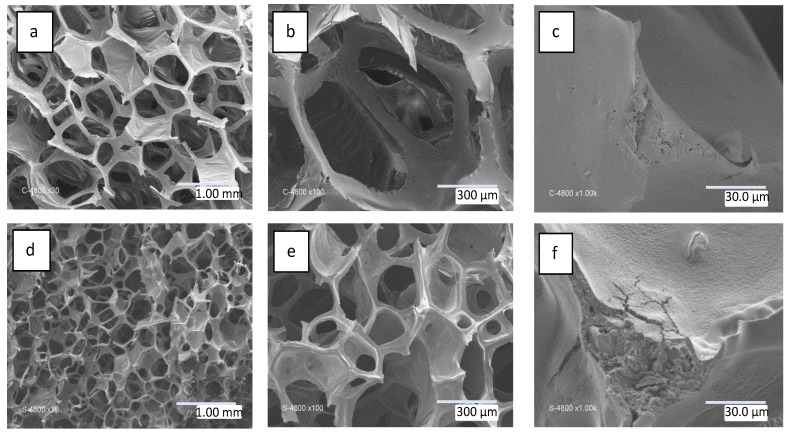
SEM images for (**a**–**c**) polyurethane foam and (**d**–**f**) SiC foam pyrolyzed at 1200 °C-2 h in Ar.

Figure 2a–c depict the open cell structure of the pristine polyurethane foam prior to the formation of SiC. The structure comprises a scaffold made of random connections of struts measuring ~500 µm and walls measuring ~0.25 mm^2^, dividing the space into interconnected open cells. Figure 2d–f show images of the foam after preceramic polymer deposition and subsequent pyrolysis. As expected, the initial structure of the PU foam is preserved, although the overall size of the open cell structure is reduced by a factor of two. The elimination and degradation of the PU template have been confirmed by submitting the pristine PU foam to the pyrolysis treatment (1200 °C-2 h in Ar). The measured weight loss was 97.45% and a brittle carbon residue was recovered.

The chemical composition of the SiC material after pyrolysis was analyzed by EDX (Figure S1). The material is primarily composed of carbon and silicon, with less than 10 at.% of oxygen, mainly originating from the PU template and possibly residual oxygen in the oven during the pyrolysis step. The high amount of carbon (~75 at.% vs. ~25% at.% of silicon) may be a result of the pyrolysis of the PU foam template, which degradation contributes to the generation of a carbon-rich SiC material.

**Figure 3:**
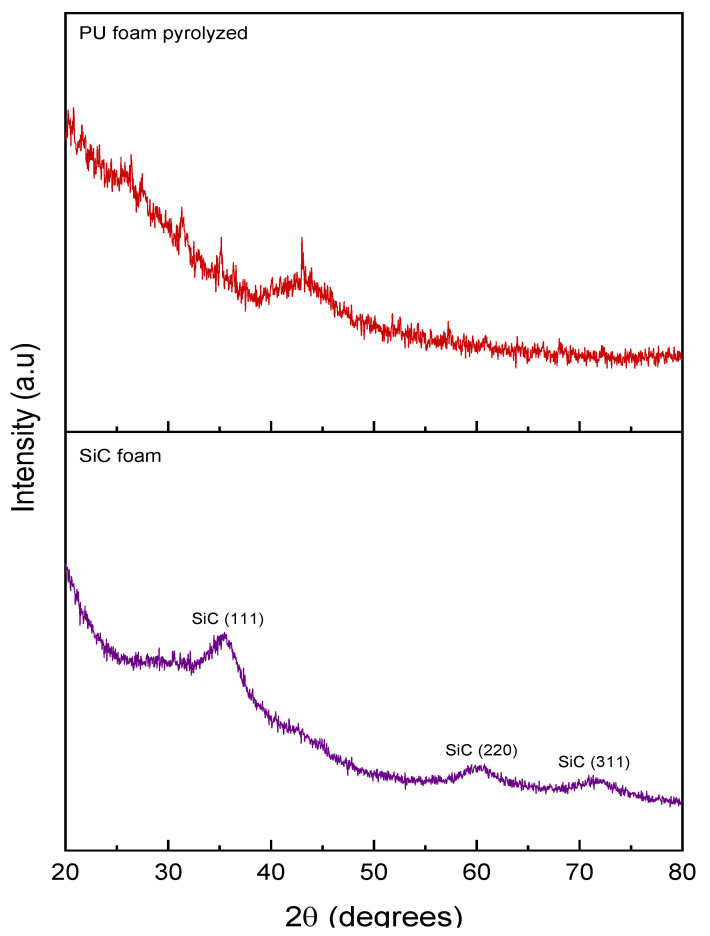
XRD patterns of SiC foam and PU foam template pyrolyzed at 1200 °C.

Figure 3 displays the XRD pattern of the SiC foam, with three diffraction peaks at 2θ = 35.5, 60.7, and 71.3°, corresponding to the (111), (220), and (311) lines of cubic SiC, respectively. The width of these peaks is relatively large, which is not surprising considering the moderate pyrolysis temperature, leading to poorly crystalline SiC [35]. The diffraction pattern of the pyrolyzed pristine PU template shows an amorphous material with a large signal around 2θ = 43° and a typical (100) line of graphite corresponding to carbon atoms in a graphenic plane. Additionally, a very broad signal around 26° is characteristic of the residue of the 002 interplanar distance in amorphous carbon [36].

**Figure 4:**
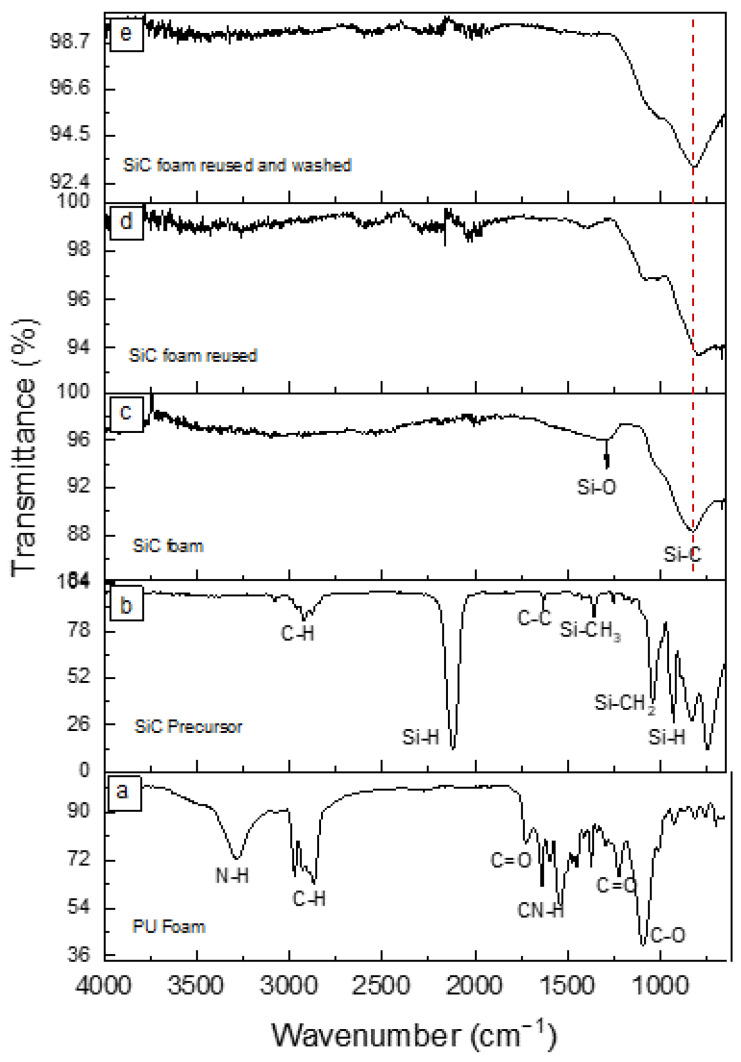
FTIR spectra for (**a**) PU foam template, (**b**) SiC precursor, (**c**) SiC foam, (**d**) SiC after photocatalytic experiment, and (**e**) SiC foam used and washed after photocatalytic experiment.

The FTIR spectrum of the PU foam template, as shown in Figure 4a, features a band at approximately 1725 cm^−1^, which corresponds to the C=O group. Additionally, the band at 1100 cm^−1^ is attributed to the C-O bond. The intensity of these two bands can be used to determine the nature of the PU foam, specifically whether it belongs to the ester- or ether-base type. In this case, the foam used belongs to the ether-type, since the intensity of the C-O bond is stronger in comparison with the C=O band [22].

A comparison of the precursor’s FTIR spectra (Figure 4b) and final SiC foam (Figure 4c) clearly demonstrates the polymer-to-ceramic conversion that occurs after the pyrolysis step. The characteristic peaks for Si-H, C-H, C-C, Si-CH_3,_ and Si-CH_2_ bonds have disappeared due to crosslinking and decomposition of organic groups, leading to the formation of a peak at ~819 cm^−1^, which corresponds to the Si-C stretching vibration [37]. The peak at ~1250 cm^−1^ is attributed to the Si-O bond. However, due to its lower intensity in comparison to the Si-C bond, it can be assumed that the SiC material has low contamination by oxygen originating from the PU foam or residual oxygen during the pyrolysis step, as previously evidenced by EDX analysis.

The FTIR spectra of the SiC foam after photocatalytic tests and after washing with ethanol and water are compared in Figure 4d,e, respectively. In Figure 4d, the emergence of a peak at 1390 cm^−1^ is attributed to the aromatic ring structures of MB [38], further confirming the adsorption of MB on the SiC foam. After the cleaning process, the spectrum in Figure 4e did not show any significant peaks related to MB and is very similar to the pristine SiC foam before use, indicating that the applied washing procedure efficiently regenerates the SiC foam and its photocatalytic properties are likely to be conserved. Interestingly, the signal coming from Si-O tends to decrease, which could reveal a reduction of surface silica species during the photocatalytic reaction as reactive oxygen species could be activated during the process [39].

**Figure 5:**
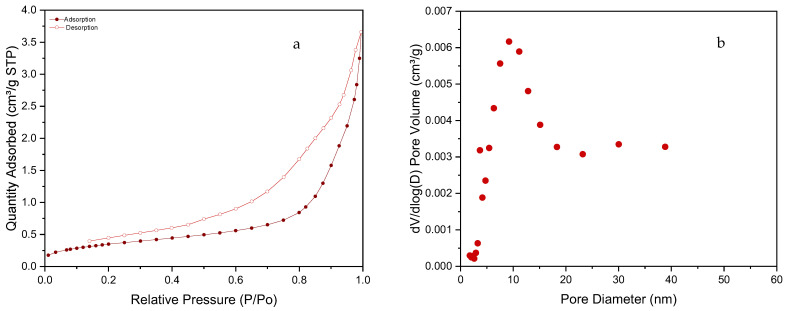
(**a**) Nitrogen adsorption-desorption isotherms of the SiC foam. (**b**) Pore size distribution of the SiC foam (BJH method desorption branch).

The nitrogen adsorption-desorption isotherms of the SiC foam are presented in Figure 5a. According to their shape and the IUPAC classification [40], it can be classified as a type IV isotherm typical for mesoporous structures. The SSA of the material was calculated by the BET method and reached values slightly above 1 m^2^/g, which is quite low, but one order of magnitude higher than values reported for SiC foams synthesized from preceramic polymer precursors by other authors [22]. Figure 5b shows the BJH pore size distribution for the SiC material. The distribution is quite large with a high contribution of mesopores with pore diameters at ~10 nm.

### 3.2. Bandgap Calculations of the Prepared SiC Foam

The bandgap for the SiC foam was calculated using the absorption spectrum fitting (ASF) method, since it has been successfully used for amorphous materials in reflection geometry [30].

The method relies on the equation linking the absorption coefficient $\alpha \left(\nu \right)$ and the optical bandgap $E_{g}$ through:

(4) $$\alpha \left(\nu \right)h\nu =B{\left(h\nu -E_{g}\right)}^{n}$$

where hν is the photon energy, B is a constant, *n* is an index that denotes the transition nature with values of *n* = 1/2 for direct allowed transition, *n* = 3/2 for direct forbidden transition, *n* = 2 for indirect allowed transition, and *n* = 3 for indirect forbidden transition. The absorption coefficient is defined as $\alpha \left(\nu \right)=\left(\frac{2.303}{d}\right)A,$ where *A* and *d* are the absorbance and the thickness of the sample, respectively. Equation (4) can also be written as a function of the wavelength (*λ*) as follows [30,41,42]:

(5) $$\alpha \left(\lambda \right)=B{\left(hc\right)}^{n-1}\lambda {\left(\frac{1}{\lambda }-\frac{1}{\lambda_{g}}\right)}^{n}$$

where $\lambda_{g}$ is the wavelength corresponding to the optical bandgap, *h* is the Plank’s constant, and *c* is the velocity of light. Then, Equation (5) can be rewritten using Beer–Lambert’s law:

(6) $$A\left(\lambda \right)=D_{1}\lambda {\left(\frac{1}{\lambda }-\frac{1}{\lambda_{g}}\right)}^{n}$$

where $D_{1}=\left[\frac{B{\left(hc\right)}^{n-1}d}{2.303}\right]$ is a constant. Thus, with Equation (6), it is possible to calculate the bandgap using $E_{g}=\frac{hc}{\lambda_{g}}=\frac{1239.83}{\lambda_{g}}$, obtaining $\lambda_{g}$ by extrapolating the linear region of the curve ${\left(\frac{A}{\lambda }\right)}^{\frac{1}{n}}vs.\left(\frac{1}{\lambda }\right)$ at ${\left(\frac{A}{\lambda }\right)}^{\frac{1}{n}}=0$.

**Figure 6:**
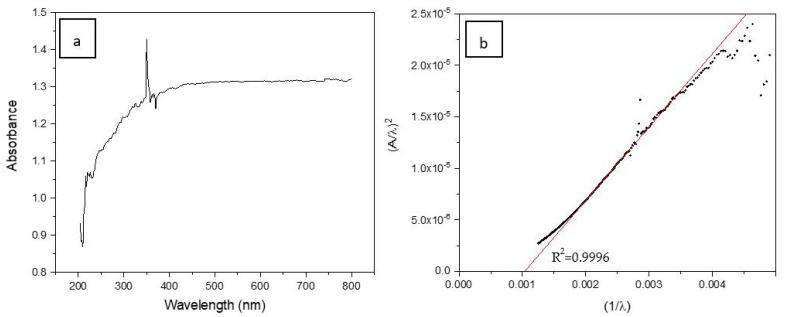
(**a**) Absorbance spectrum for SiC foam and (**b**) correlation (*A*/*λ*)^1/*n*^ vs. 1/λ (*n* = 1/2).

Figure 6a shows the absorbance spectrum for the studied SiC foam. The bandgap was calculated by tracing the curve (*A*/*λ*)^1/*n*^ vs. 1/*λ* with different n values. These plots are shown in supplementary information in Figure S2. It was evidenced that the best fitting was achieved for *n* = 1/2 (Figure 6b), with the linear correlation coefficient *R*^2^ = 0.9996, indicating a direct allowed transition. The bandgap obtained by the ASF method is equal to 1.29 eV (962 nm), i.e., slightly larger than that of silicon (1.18 eV) [43]. Considering that the material could be excited with a wavelength equal or higher than this value, it can be assumed that the SiC foam could work as an active photocatalyst under visible light irradiation.

### 3.3. Adsorption Capacity and Photocatalytic Performance of SiC Foams under Visible Light Irradiation

**Figure 7:**
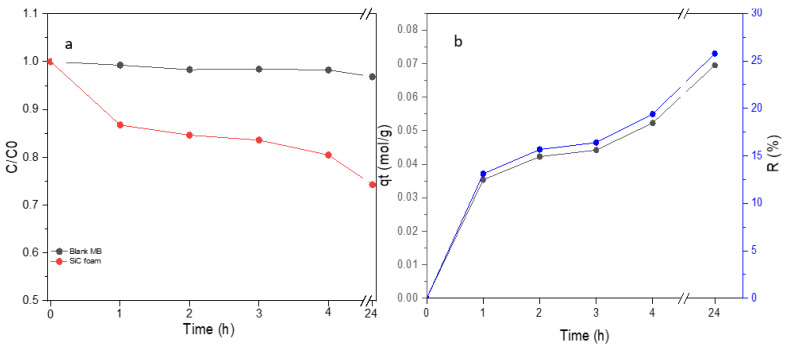
(**a**) Evolution of MB concentration in the solution with and without the SiC catalyst (in absence of light irradiation), and (**b**) mass percentage R of MB retained by the SiC foam (blue curve) and adsorption capacity (mol/g) for the SiC foam (black curve).

The adsorption capacity of the SiC foam was evaluated by immersing it in an MB solution for 4 h in the absence of light irradiation. A control experiment was also conducted, where a pure MB solution was left in the dark without the SiC foam. The evolution of MB concentration in the solution without any catalysts and the solution containing the SiC foam is shown in Figure 7a. In the absence of light irradiation and without SiC foam, the MB does not undergo any significant degradation over time. However, in the presence of the SiC foam, an important decrease in MB concentration is evidenced at the beginning of the experiment before reaching semi-equilibrium with a very slow rate of MB concentration decrease. This result thus suggests a non-negligible adsorption capacity of the SiC foam itself. The amount of MB adsorbed per gram of foam at a specific time *t* can be easily derived from Equation (2), as shown in Figure 7b (black curve) with the plot of *q_t_* vs. *t*. Moreover, from Equation (3), one can also quantify the evolution of the MB % present on the foam as plotted in Figure 7b (blue curve). The high adsorption rate at the beginning of the experiment results from a high number of available sorption sites. The progressive occupation of vacant adsorption sites [44] results in a deceleration of MB concentration decrease.

The adsorption kinetics of the SiC foam were analyzed using two models, the pseudo-first order (PFO) and the pseudo-second order (PSO) kinetic models. These models are commonly employed to analyze time-dependent adsorption data [45]. During the adsorption process, both physical (physisorption) and chemical (chemisorption) interactions might exist between the adsorbent (SiC foam) and the adsorbate (MB) [8]. The PFO model describes a physisorption mechanism while the PSO one describes a chemisorption.

**Figure 8:**
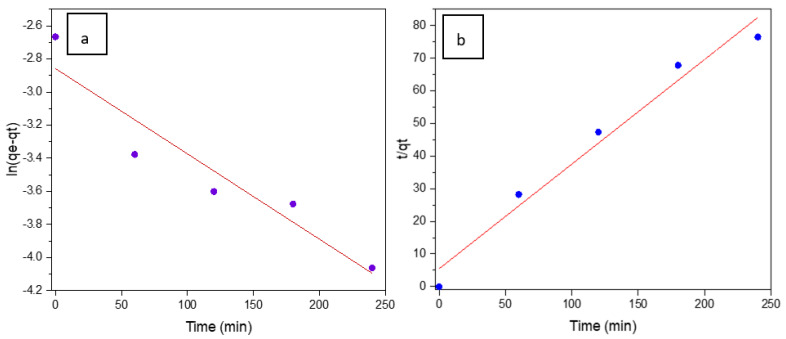
(**a**) PFO plot (ln(*q_e_* − *q_t_*) vs. *t*, (**b**) PSO plot *t*/*q_t_* vs. *t*, for the SiC foam.

The pseudo first-order equation of Lagergren [46] in a linear way is represented by Equation (7):

(7) $$ln(q_{e}-q_{t})=l{n(q}_{e})-\frac{k_{1}}{2.303}t$$

where *q_e_* and *q_t_* are the adsorption capacities at equilibrium and at time *t*, respectively. The value of $k_{1}$ (0.0051 min^−1^, with $R^{2}=0.8973$) was obtained from the slope of the linear fitting of the plot of $ln(q_{e}-q_{t})$ vs. *t*, shown in Figure 8a.

For the PSO kinetic model [47], Equation (8) was used:

(8) $$\frac{t}{q_{t}}=\frac{1}{k_{2}q_{e}^{2}}+\frac{t}{q_{e}}$$

where $k_{2}$ (g/mg min) is the PSO rate constant. The plot of $\frac{t}{q_{t}}$ vs. t was used to obtain the rate constant $k_{2}$ [48]. The plot is shown in Figure 8b, and from the slope, $k_{2}$ was determined to be 0.321 min^−1^ with a correlation coefficient $R^{2}=0.9707$.

From the plots in Figure 8a,b and comparing the correlation coefficients $R^{2}=0.8973$ for PFO and $R^{2}=0.9707$ for PSO, it could be assumed that the PSO kinetic model dominates the adsorption reaction. Hence, chemisorption is the predominant mechanism in the studied adsorption experiments [44] as already observed in porous polymer-derived SiOC aerogels described in the literature [49].

Measurements of the photocatalytic degradation of MB were conducted in the configuration depicted in Figure 1. The details concerning the photocatalytic degradation mechanism of MB in a water environment can be found elsewhere [2], as well as the generation of active oxidation species by semiconductor catalysts [16,17,50,51,52]. The scope of this study was to assess the performance and potential use of the prepared SiC-based foams in comparison with other photocatalytically active materials.

**Figure 9:**
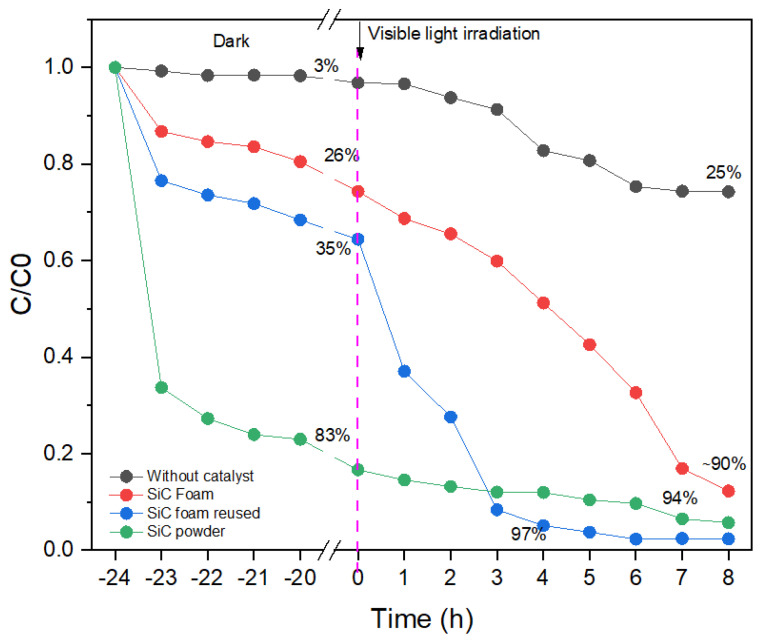
Evolution of the MB concentration under different experimental conditions.

The results of photocatalytic experiments are shown in Figure 9, along with data obtained in the absence of light irradiation or catalyst (SiC foam). When examining the experiments with the MB solution in absence of a catalyst, it was observed that the slight decrease in MB concentration (~3%) during the dark period reaches ~25% after 8 h under visible light irradiation. This decrease in MB concentration is mainly attributed to the photolysis effect induced by visible light. In the presence of SiC foam, the decrease of the MB concentration is significantly higher: from ~26% in the dark period (adsorption effect) up to 62% in the presence of light irradiation (photocatalytic activity), thus yielding MB elimination of about 88% at the end of the experiment.

For comparative purposes, similar experiments were also conducted with the same amount of SiC in powder form (grounded SiC foam). As observed in Figure 9 (green curve), the adsorption capacity of the SiC powder was significantly higher. This observation was not surprising, as the accessible active surface area of the SiC fine powder is higher in comparison with the SiC foam monolith of the same composition. On the other hand, the powder material was quickly saturated, and the photocatalytic effect was significantly reduced, resulting in limited photocatalytic degradation of MB molecules. In fact, the predominant mechanism in such a powder system is mainly based on simple adsorption of the undesirable molecules, with only limited abatement by photocatalytic degradation. This adsorption-based abatement mechanism is not very efficient as the removal capacity of the SiC powder material is limited by surface saturation. Additionally, the regeneration of the sorbent/catalyst requires tedious powder manipulation and possible loss of material during the washing and filtration process. On the contrary, the manipulation of the robust SiC foam monoliths and their regeneration is significantly easier, allowing for their repetitive use and cycling. The activity of the recycled catalyst is shown in Figure 9 (blue curve). Interestingly, it has been observed that the recycled SiC-foam features better absorption efficiency followed by higher photocatalytic activity compared with original (as-prepared) foams. This phenomenon is most likely related to the progressive hydroxylation of the SiC material increasing its hydrophilicity [53,54], which might favor/enhance the contact between the aqueous solution and the catalyst surface. This observation thus confirms the attractive properties of SiC-foams for the photocatalytic removal of organic molecules in water media and their potential application in contactor devices working in a semi-continuous mode.

It can be observed (as shown in Table 1) that the optimal SiC foams offer MB degradation efficiencies that are comparable to those of other photocatalysts tested in the visible light region. Furthermore, considering the advantage of the relatively simple character of the protocol for producing uniform SiC foams, this material emerges as a highly competitive new photocatalytic material for application under visible light irradiation.

For the further valorization of this material, we are currently working on the optimization of the design of these SiC foams for use as catalytic contactors for reactors operating in semi-continuous mode (alternated periods of photocatalytic reaction and catalyst regeneration). This strategy requires maximizing the contact of the treated solution with the SiC pore surface while also ensuring the highest possible surface area accessible for light illumination. To achieve this, it is essential to consider not only the optimization of the SiC foam porous structure, but also its geometric shape and size, with sufficient mechanical strength to prevent any possible foam disintegration during the continuous flow of the liquid through the catalytic contactor thickness. The degradation kinetics will be improved by controlling both the SiC foam microstructure and its surface composition/decoration. Finally, this promising SiC functional material is also being considered for shaping multifunctional membranes (a thin mesoporous layer on top of support) that combine both photocatalytic activity and separation effects during the filtration process.

## 4. Conclusions

SiC foams were fabricated, using a PU template and AHPCS as the SiC precursor, through the replica method in the presence of a triblock copolymer as a porogen. During the pyrolysis step, the PU template was removed, resulting in a final SiC-based material that retained the initial PU foam geometry with cell sizes reduced by a factor of two. The as-prepared SiC foam was successfully tested as a photocatalyst for the degradation of MB molecules (model organic pollutant) under visible light irradiation. The adsorption capacity of the SiC foam was also analyzed in the absence of light, showing an overall increase in the adsorption capacity and accessible surface area. Thanks to the induced photocatalytic reaction, one could reach over 90% of MB removal under optimal experimental conditions. Moreover, it has been verified that the SiC foams could be regenerated by a simple washing process without having to apply any thermal or harsh chemical treatment, and after recycling, the photocatalytic performance was retained. These results offer further perspectives for the production of efficient reusable semi-continuous water purification units (catalytic contactors or membrane reactors) with optimally shaped SiC photocatalysts working under the effect of visible (solar) light irradiation.

## Figures and Tables

**Table 1:** Comparison of performance for different photocatalysts under visible light irradiation.

Photocatalyst [ref]	Bandgap (eV)	Efficiency for MB Abatement
SiC nanowires [28]	-	96% after 360 min
SiC hollow spheres [29]	2.15	98% after 300 min
PVP-capped ZnS [32]	4.07	81% after 360 min
Er_2_O_3_-coated silicon nanowires [52]	-	98% after 120 min
SiC foams (this work)	1.29	88% after 480 min

## Data Availability

The data presented in this study are available on request from the corresponding author. The data are not publicly available due to data protection politics of the IEM.

## Supplementary Materials

The following supporting information can be downloaded at: https://www.mdpi.com/article/10.3390/ma16041328/s1, Figure S1. EDX pattern for the SiC foam. Figure S2. Linear fitting for (A/λ)^1/n^ vs. 1/λ with (a) n = 2, (b) n = 3/2, (c) n = 3 and (d) n = 1/2.
