# Supplementary material for: SiC Foams for the Photocatalytic Degradation of Methylene Blue under Visible Light Irradiation
Source: Materials (Basel). 2023 Feb 4;16(4):1328. doi: 10.3390/ma16041328 (PMC9959366; doi:10.3390/ma16041328)
Supplement: Supplementary file 1 [file materials-16-01328-s001.zip › materials-2186154-supplementary.pdf]

Supplementary information

SiC foams for the photocatalytic degradation of organic pollutants under visible light irradiation

K.B. Cervantes-Diaz, M. Drobek, A. Julbe, and J. Cambedouzou*

Institut Européen des Membranes (IEM), Univ Montpellier, CNRS, ENSCM, Place Eugene Bataillon; 34095 Montpellier, France.

*Corresponding: julien.cambedouzou@enscm.fr

Figure S1 shows the EDX spectrum and the associated composition table of the SiC foam.

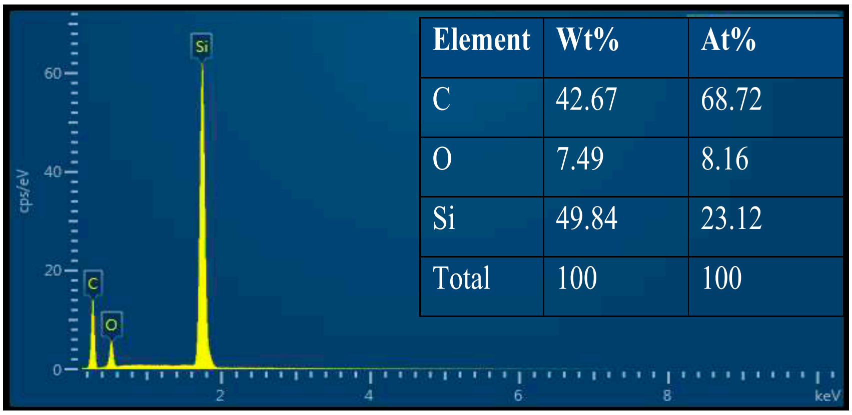

Element	Wt%	At%
C	42.67	68.72
O	7.49	8.16
Si	49.84	23.12
Total	100	100

Figure S1. EDX pattern for the SiC foam.

Figures S2 a) to d) show the linear fit for the bandgap calculations using the ASF method. It was observed that the best fitting occurred for n=1/2 (figure 1 d), with the linear correlation coefficient R^2=0.9996, indicating a direct allow transition.

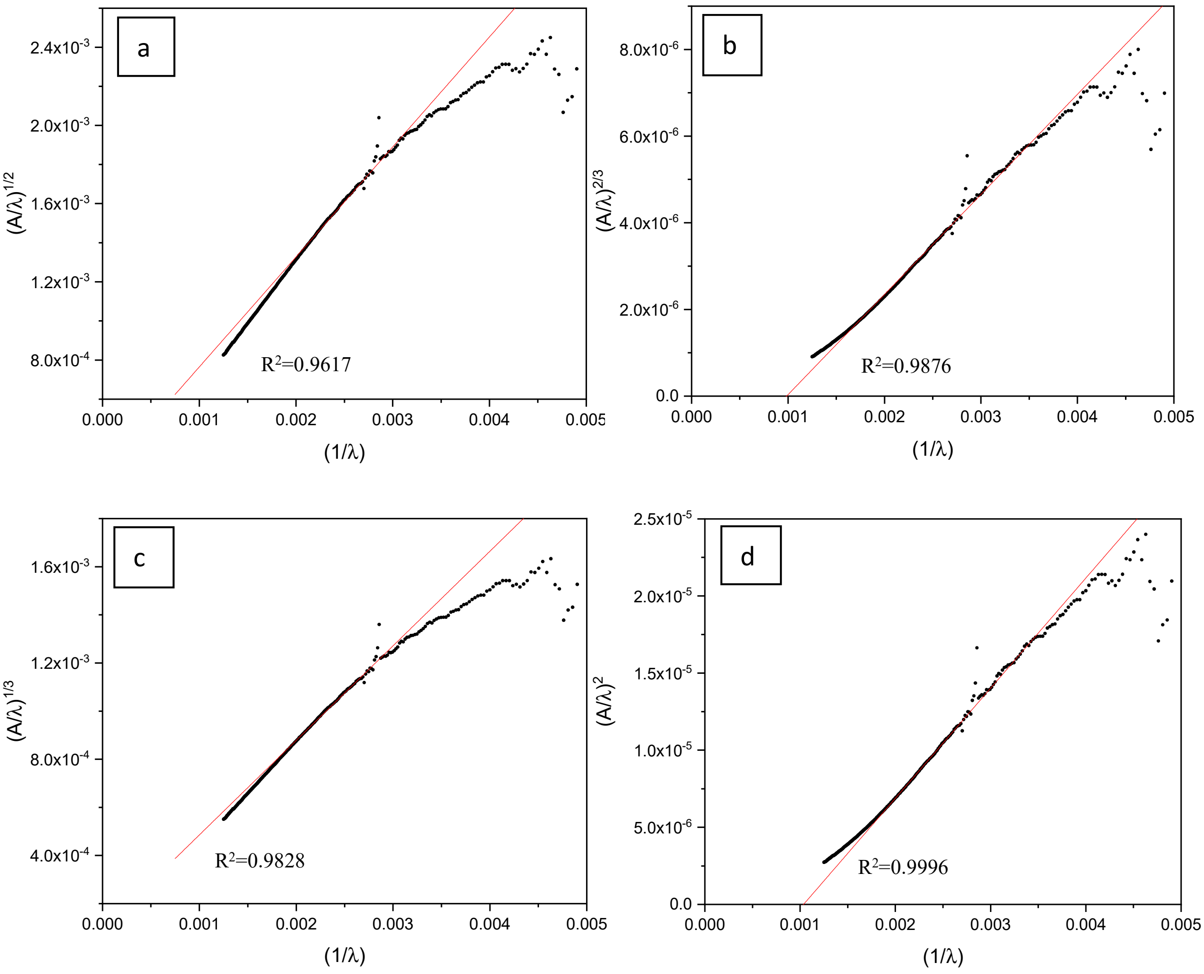

Figure S2. Linear fitting for $(A/\lambda)^{1/n}$ vs $1/\lambda$ with a) n=2, b) n=3/2, c) n=3 and d) n=1/2.
